# Predicting emotional disturbance in breast cancer patients: The role of self-empowerment skills, interpersonal relationships, and demographic factors

**DOI:** 10.1192/j.eurpsy.2025.1786

**Published:** 2025-08-26

**Authors:** M. Firoozi, M. Sharifi, C. A. Lewis

**Affiliations:** 1 Psychology, University of Tehran, Tehran; 2 Psychology, Islamic Azad University, Bandargaz Branch, Bandargaz, Iran, Islamic Republic Of; 3 Psychology, Leeds Trinity University, Leeds, United Kingdom

## Abstract

**Introduction:**

Breast cancer represents a significant health concern among Iranian women, notably impacting their mental health. This has spurred considerable interest within health psychology, given breast cancer’s profound effects on both physical and psychological well-being.

**Objectives:**

This study aimed to explore the predictability of emotional disturbances through psychological, socio-cognitive variables, and to quantify their relative impacts among breast cancer patients.

**Methods:**

Employing a descriptive and exploratory approach, this research involved 736 breast cancer patients aged 19 to 80 from Shahada Tajrish Hospital, Tehran during the first eight months of 2017. Participants were selected and screened based on specific inclusion and exclusion criteria. Measures included the Self-Empowerment Skills Scale, the Quality of Interpersonal Interactions Scale, and the Emotional Disturbance Scale. An emotion management therapy program was offered to motivate patient participation.

**Results:**

Analysis revealed significant associations between emotional disturbance and factors such as self-empowerment skills, quality of interpersonal interactions, and various demographic variables including age, education level, employment status, household headship, and gender. Notably, interpersonal quality and self-empowerment skills emerged as the most influential predictors.

**Conclusions:**

The findings underscore the critical roles of interpersonal relationships, self-empowerment skills, and demographic characteristics in influencing the emotional well-being of breast cancer patients. It is recommended that psychologists emphasize these factors when assessing and promoting the mental health of this population.

**Disclosure of Interest:**

None Declared

